# Antidepressant-like effects of psychedelics in a chronic despair mouse model: is the 5-HT_2A_ receptor the unique player?

**DOI:** 10.1038/s41386-024-01794-6

**Published:** 2024-01-11

**Authors:** Mehdi Sekssaoui, Joël Bockaert, Philippe Marin, Carine Bécamel

**Affiliations:** Institut de Génomique Fonctionnelle, Université de Montpellier, Centre National de la Recherche Scientifique, Institut National de la Santé et de la Recherche Médicale, F-34094 Montpellier, France

**Keywords:** Depression, Anxiety

## Abstract

Major depressive disorder (MDD) is one of the most disabling psychiatric disorders in the world. First-line treatments such as selective serotonin reuptake inhibitors (SSRIs) still have many limitations, including a resistance to treatment in 30% of patients and a delayed clinical benefit that is observed only after several weeks of treatment. Increasing clinical evidence indicates that the acute administration of psychedelic agonists of the serotonin 5-HT_2A_ receptor (5-HT_2A_R), such as psilocybin, to patients with MDD induce fast antidepressant effects, which persist up to five weeks after the treatment. However, the involvement of the 5-HT_2A_R in these antidepressant effects remains controversial. Furthermore, whether the hallucinogenic properties of 5-HT_2A_R agonists are mandatory to their antidepressant activity is still an open question. Here, we addressed these issues by investigating the effect of two psychedelics of different chemical families, DOI and psilocybin, and a non-hallucinogenic 5-HT_2A_R agonist, lisuride, in a chronic despair mouse model exhibiting a robust depressive-like phenotype. We show that a single injection of each drug to wild type mice induces anxiolytic- and antidepressant-like effects in the novelty-suppressed feeding, sucrose preference and forced swim tests, which last up to 15 days. DOI and lisuride administration did not produce antidepressant-like effects in 5-HT_2A_^−/−^ mice, whereas psilocybin was still effective. Moreover, neither 5-HT_1A_R blockade nor dopamine D_1_ or D_2_ receptor blockade affected the antidepressant-like effects of psilocybin in 5-HT_2A_^−/−^ mice. Collectively, these findings indicate that 5-HT_2A_R agonists can produce antidepressant-like effects independently of hallucinogenic properties through mechanisms involving or not involving the receptor.

## Introduction

Major depressive disorder (MDD) is one of the leading causes of disability worldwide. It can affect up to 20% of people during the entire lifespan in developed countries, generating high economic burdens [1]. MDD prevalence is increasing since the COVID-19 pandemics that has left more people in mental distress than ever before. MDD strongly compromises the quality of life of patients and can ultimately lead to suicide, the most dramatic outcome contributing to the high mortality of MDD [1, 2]. Conventional antidepressants, such as selective serotonin reuptake inhibitors (SSRIs), show unequivocal therapeutic efficacy, but several weeks of treatment are generally required to observe a clinical benefit. Furthermore, less than 50% of patients show complete remission even with optimized therapies, and 30% are resistant to any treatment. There is thus an urgent need to develop fast-acting and more efficient antidepressants for patients with MDD, especially for those with suicidal ideations or behaviors.

A recent paradigm shift in the treatment of MDD has been the use of the NMDA receptor antagonist ketamine which produces fast antidepressant effects even in patients resistant to conventional antidepressants. In fact, ketamine antidepressant-like effects in preclinical models of depression are independent of NMDA receptor inhibition but require AMPA receptor activation [3]. Serotonergic psychedelics, such as Lysergic Acid Diethylamide (LSD) or psilocybin have also been recognized for their potential to treat MDD [4–7]. Clinical trials, generally of small sizes, revealed that one or two doses of psilocybin induce fast (within hours) and prolonged alleviation of depressive symptoms that last several weeks and, sometimes, up to 12 months after the treatment [8, 9]. A larger phase 2b clinical trial in patients with major depressive episodes and resistant to classical treatments conducted in 10 countries in Europe and North America recently showed that 30% of patients who received a single dose of psilocybin (25 mg) in combination with psychological support, had a rapid reduction of depressive symptoms that persisted three weeks after the treatment [10]. Furthermore, unlike ketamine, psychedelics do not cause dependence or withdrawal syndrome [11] and induce side effects (anxiety, alteration of perception, or psychotic states) only in a small proportion of patients [12], thus raising a great hope for the treatment of patients with MDD associated to suicidal ideations/behaviors.

Although there is a consensus that 5-HT_2A_ receptor (5-HT_2A_R) activation mediates the psychedelic effects of LSD and psilocybin in human and mice [13–16], its role in their antidepressant effects remains controversial [17, 18]. Another open question is whether the psychedelic properties of 5-HT_2A_R agonists are necessary for their antidepressant activity. Here, we investigated these issues in the chronic despair model (CDM) in the mouse, a preclinical model of depression of high translational value. We show that the 5-HT_2A_R is involved in the antidepressant-like effects of DOI, a synthetic psychedelic, but not in the effects of psilocybin. We also show that lisuride, a non-hallucinogenic ergot derivative that behaves as a 5-HT_2A_R agonist, induces antidepressant-like effects in the same model, suggesting that the antidepressant activity of 5-HT_2A_R agonists is independent of their psychedelic properties.

## Materials and methods

### Animals

Wild‐type male and female C57BL/6J mice were purchased from Charles River Laboratories. 5-HT_2A_R-deficient (5‐HT_2A_^−/−^) mice, originally generated at Columbia University, were kindly provided by Dr Laurence Lanfumey, Institut de Psychiatrie et Neurosciences de Paris (IPNP), 102-108 rue de la santé, 75014 Paris, France. These mice were originally generated on a 129S6/SvEv background and backcrossed over at least ten generations onto the inbred C57BL/6J line. Mice from both sexes were indifferently used in behavioral experiments. Mice were housed under standardized conditions with a 12‐h light (from 7:00 am to 7:00 pm)/dark cycle, stable temperature (22 ± 1 °C), controlled humidity (55 ± 10%), and free access to food and water. Animal husbandry and experimental procedures were performed in compliance with the animal use and care guidelines of the University of Montpellier, the French Agriculture Ministry, and the European Council Directive (86/609/EEC). All the experiments were conducted with animals aged from postnatal day 60.

### Drugs and treatments

2,5-Dimethoxy-4-iodoamphetamine (DOI; Sigma D101), psilocybin (Cayman Chemical No.14041) and Lisuride maleate (Santa Cruz Biotechnologies) were injected intraperitoneally at the dose of 1 mg/kg. WAY-100635 (Sigma W108) was injected at the dose of 0.5 mg/kg. Ketamine (Imalgene 1000) was injected at the dose of 3 mg/kg. Eticlopride (Tocris 1847) and SCH-23390 (Tocris 0925) were injected sub-cutaneously at the dose of 0.03 mg/kg. For microdosing experiments, DOI and psilocybin were injected daily at the dose of 0.05 mg/kg that does not induce head twitch response (HTR) in mice, during 6 days. DOI, WAY-100635 Ketamine, eticlopride and SCH-23390 were dissolved in saline solution (0.9% NaCl). Psilocybin and Lisuride were first dissolved in DMSO and then diluted in saline solution (DMSO 1.5%, 0.9% NaCl). Vehicle groups received this solution. Treatments were given in the morning, between 9:00 and 10:00 am. A volume of 5 mL/kg was administrated for each drug.

### Behavioral tests

#### Chronic behavioral despair model

In order to induce depressive-like symptoms in mice, we used a previously well-described protocol [19, 20] where mice were subjected to repeated swimming in a 2-liter beaker containing 20 cm of water (22–25 °C) for 10 min daily during 5 consecutive days (induction phase). Then mice were kept in their home cages without any stress (resting phase). Three days after the last swimming session (Day 0), mice were injected either with vehicle, or DOI (1 mg/kg, i.p.), or psilocybin (1 mg/kg, i.p.), or psilocybin (1 mg/kg, i.p.) + WAY-100635 (0.5 mg/kg, i.p.) or lisuride (1 mg/kg, i.p.) or psilocybin (1 mg/kg, i.p.) + eticlopride (0.03 mg/kg, s.c.), or psilocybin (1 mg/kg, i.p.) + SCH23390 (0.03 mg/kg, s.c.), or ketamine (3 mg/kg, i.p.). All experiments (induction and tests) were performed in the morning. For microdosing experiments, mice were daily injected either with vehicle, or DOI (0.05 mg/kg, i.p.) or psilocybin (0.05 mg/kg, i.p.) during 6 days. The first behavioral test was performed 48 h after the last injection of psychedelics.

#### Head twitch responses

HTRs were monitored over 30 min after the administration of either vehicle, DOI (1 mg/kg, i.p.) or psilocybin (1 mg/kg, i.p.) or lisuride (1 mg/kg, i.p.) or DOI (0.05 mg/kg, i.p.) or psilocybin (0.05 mg/kg, i.p.) in both naive C57BL/6 J and 5-HT_2A_^−/−^ mice. Data were categorized in 10-min time frames (0–10 ; 10–20 ; 20–30 min). HTR was counted as a head flick by a blind observer.

#### Novelty-suppressed feeding test

The test was conducted in a 50 cm-large square open field, the floor of which was covered with approximately 2 cm of wooden bedding. Twenty-four hours prior to behavioral testing, the food was removed from the home cage. At the time of testing, a single pellet of food was placed on a white paper in the center of the box. High illumination (1000 lux) was used to produce an anxiogenic environment. Each mouse was placed in the same corner of the open field and a stopwatch was immediately started. The latency to eat (defined as the mouse biting the pellet) was measured. The testing sessions lasted 10 min. The maximum value (600 s) was attributed to animals that do not bite the pellet during the testing session. Immediately after biting the food pellet, mice were placed in a test cage containing a new, weighed food pellet. The amount of pellet eaten for 5 min was measured. Then, the animals were placed back in their home cage with food and water *ad libitum* [21, 22].

#### Forced swimming test

The procedure used was adapted from Porsolt et al. [23]. Mice were placed in a 2-L beaker filled with 20 cm of 23–25 °C water and videotaped for the entire session (6 min). Immobility duration was scored for the entire session by a blinded observer.

#### Sucrose preference test

The protocol used was adapted from Liu et al. [24, 25]. During the first phase, mice in their home cages were continuously offered two bottles, one containing a sucrose (1%) solution and one containing regular water, for 48 h. The position of the two bottles in each cage was swapped every 12 h to avoid place preference bias. Mice also had access to food *ad libitum*. They were then transferred to the testing cage for 12 h with food and water *ad libitum* for adaptation. Thereafter, they were deprived of both food and water for 24 h. Immediately after deprivation, they were offered the bottle of 1% sucrose solution and the bottle of regular water for 12 h. Each bottle was weighed before and after the test. No significant differences in total volume intake (~10 ml) were measured between the groups. At the end of the test, animals were placed again in the home cage with food and water *ad libitum*. The sucrose preference index was defined as (sucrose intake – water intake)/total intake [18, 26]. Cages with leaking bottles were excluded.

#### Circular corridor (cyclotron)

Locomotor activity (number of beam interruptions) was measured in a circular corridor (14-cm wide, 18-cm diameter) with four infrared beams placed at 90° angles (Imetronic, Pessac, France) in a low luminosity environment. Mice activity was monitored for 30 min.

### Statistical analysis

Data are mean values ± SEMs. Statistical analysis was performed using Graphpad Prism (v. 9.5.0). Outliers were removed from data analysis using Graphpad Prism. The threshold for statistical significance was set at 0.05. In each dataset, Gaussian distribution was verified (Shapiro–Wilk normality test), and the homogeneity of sample variance was tested using Bartlett’s test. As long as no variance among groups was detected, ordinary one-way ANOVA test was performed. Data were analyzed by one-way ANOVA or two-way ANOVA and Dunnett’s adjustment for post-hoc analysis in groups with normal distribution and by Kruskal–Wallis test and Dunn’s adjustment for post-hoc analysis in groups with non-parametric distribution.

## Results

### Antidepressant-like effects of 5-HT_2A_R agonists in the chronic despair mouse model of depression

**Fig. 1:**
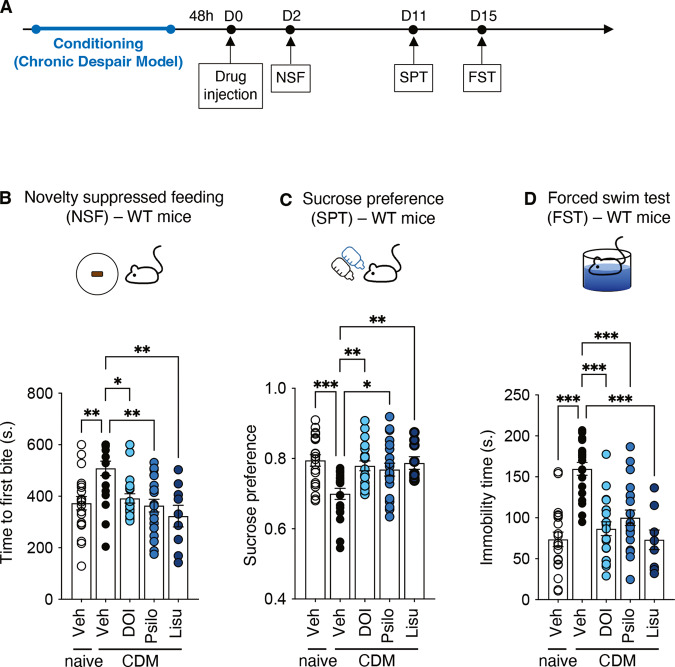
Hallucinogenic or non-hallucinogenic 5-HT_2A_R agonists produce antidepressant-like effects in CDM mice. **A** Timeline of treatment and behavioral experiments in wild type (WT) CDM mice. Mice received an injection of the tested compounds 48 h after the end of the chronic despair protocol. They were then subjected to tests assessing Novelty Suppressed Feeding (NSF), Sucrose Preference (SPT) and Forced Swimming (FST) from 2 to 15 days after the injection of the drug (Day 0). **B** The histogram represents the time of first bite (seconds) measured in the NSF paradigm for each condition. Naive mice + vehicle (*n* = 20), CDM mice + vehicle (*n* = 19), CDM mice + DOI (*n* = 20), CDM mice + psilocybin (*n* = 20), CDM mice + lisuride (*n* = 9). *p < 0.05, **p < 0.01 *vs*. CDM condition, Kruskal–Wallis test followed by Dunn’s test. **C** The histogram represents the sucrose preference index calculated for each condition. Naive mice + vehicle (*n* = 20), CDM mice + vehicle (*n* = 19), CDM mice + DOI (*n* = 20), CDM mice + psilocybin (*n* = 20), CDM mice + lisuride (*n* = 9). *p < 0.05, **p < 0.01, ***p < 0.001 *vs*. CDM condition, one-way ANOVA test followed by Dunnett’s test. **D** The histogram represents the immobility time (seconds) measured in the FST for each condition. Naive mice + vehicle (*n* = 20), CDM mice + vehicle (*n* = 19), CDM mice + DOI (*n* = 20), CDM mice + psilocybin (*n* = 20), CDM mice + lisuride (*n* = 9). ***p < 0.001 *vs*. CDM condition, one-way ANOVA test followed by Dunnett’s test. See Table 1 for mean ± SEM values.

To investigate the antidepressant-like effects of 5-HT_2A_R agonists, CDM mice were consecutively subjected to behavioral tests that collectively evaluate depressive-like behaviors, namely the novelty-suppressed feeding test (NSF, [21, 22]) to assess anxiety-like behavior, the sucrose preference test (SPT) to assess anhedonia [24, 25] and the forced swim test (FST) to assess despair behavior [23]. These tests were performed from 2 to 15 days after a single intraperitoneal injection of either vehicle or a 5-HT_2A_R agonist (Fig. 1A and Table 1). Two hallucinogenic 5-HT_2A_R agonists belonging to different chemical families, psilocybin (1 mg/kg), a tryptamine and 2,5-Dimethoxy-4-iodoamphetamine (DOI, 1 mg/kg), a phenethylamine, were tested. We also investigated the effect of lisuride (1 mg/kg), a non-hallucinogenic 5-HT_2A_R agonist prescribed for more than 40 years for its anti-parkinsonian activity and ability to lower prolactin level [27, 28]. We first verified that administration of DOI or psilocybin induced a significant increase in HTR in wild type mice, compared to vehicle-injected animals, demonstrating hallucinogenic-like activity, whereas administration of lisuride had no effect (Supplementary Fig. S1A and Table 1). As expected, administration of DOI or psilocybin to 5-HT_2A_^−/−^ mice did not increase the number of head twitches (Supplementary Fig. S1B and Table 1). CDM mice exhibited a strong depressive-like phenotype, as assessed by their increase in latency to feed, decrease in sucrose consumption and increase in immobility time in the FST, compared with non-conditioned mice (naive mice, Fig. 1B–D and Table 1). Administration of either DOI or psilocybin to CDM mice induced a significant decrease in the latency to feed in the NSF test and in the immobility time in the FST, compared to vehicle-injected mice (Fig. 1B, D and Table 1). DOI and psilocybin administration to CDM mice also restored sucrose preference to a level similar to that measured in non-conditioned mice (Fig. 1C and Table 1). Similar results were obtained in lisuride-injected CDM mice (Fig. 1B–D and Table 1) and in CDM mice injected with ketamine (3 mg/kg, i.p.) used as a reference molecule producing fast antidepressant action [20] (Supplementary Fig. S2A–C and Table 1). None of these treatments affected locomotor activity of mice, assessed by the number of beam interruptions in the cyclotron test (Supplementary Fig. S3 and Supplementary Table S1). Neither DOI, nor psilocybin nor lisuride administration induced a significant effect in non-conditioned mice in the three behavioral tests (Supplementary Fig. S4 and Table 1). Collectively, these results indicate that hallucinogenic 5-HT_2A_R agonists as well as a 5-HT_2A_R agonist devoid of psychedelic activity in rodents [15, 29] and humans [28, 30] induce rapid and prolonged antidepressant-like effects in CDM mice.

**Table 1:** Mean ± SEM, *n* and *p* values related to Figs. 1–4 and Supplementary Figs. S1, S2, S4 and S5.

Figure number	Test	*n*	Mean ± SEM	Exact *p* value	F/H/W value
1B	Kruskal–Wallis + Dunn’s multiple comparisons	20 (10M/10F)	373 ± 26		H = 18,16
		19 (10M/9F)	508 ± 28	*p* = 0.005	
		20 (11M/9F)	392 ± 18	*p* = 0.015	
		20 (10M/10F)	364 ± 25	*p* = 0.002	
		9 (5M/4F)	323 ± 42	*p* = 0.003	
1C	One-way ANOVA + Dunnett’s multiple comparisons	20 (10M/10F)	0.795 ± 0.016		F (4, 83) = 5.473
		19 (10M/9F)	0.700 ± 0.016	*p* < 0.001	
		20 (11M/9F)	0.779 ± 0.014	*p* = 0.002	
		20 (10M/10F)	0.769 ± 0.018	*p* = 0.010	
		9 (5M/4F)	0.787 ± 0.018	*p* = 0.010	
1D	One-way ANOVA + Dunnett’s multiple comparisons	20 (10M/10F)	74 ± 9		F(4, 83) = 15.56
		19 (10M/9F)	160 ± 8	*p* < 0.001	
		20 (11M/9F)	87 ± 8	*p* < 0.001	
		20 (10M/10F)	100 ± 9	*p* < 0.001	
		9 (5M/4F)	73 ± 12	*p* < 0.001	
2A	Kruskal–Wallis + Dunn’s multiple comparisons	19 (10M/9F)	354 ± 24		H = 35,19
		17 (8M/9F)	565 ± 11	*p* < 0.001	
		18 (10M/8F)	535 ± 23	*p* > 0.999	
		8 (4M/4F)	598 ± 1	*p* > 0.999	
2B	Kruskal–Wallis + Dunn’s multiple comparisons	19 (10M/9F)	0.819 ± 0.015		H = 11,74
		19 (10M/9F)	0.709 ± 0.024	*p* = 0.003	
		19 (10M/9F)	0.762 ± 0.028	*p* = 0.182	
		9 (4M/5F)	0.749 ± 0.029	*p* > 0.999	
2C	One-way ANOVA + Dunnett’s multiple comparisons	19 (10M/9F)	80 ± 7		F (3, 62) = 29.22
		19 (10M/9F)	173 ± 11	*p* < 0.001	
		19 (10M/9F)	185 ± 9	*p* = 0.717	
		9 (4M/5F)	186 ± 9	*p* = 0.779	
3A	Kruskal–Wallis + Dunn’s multiple comparisons	19 (10M/9F)	354 ± 24		H = 33,36
		17 (8M/9F)	565 ± 11	*p* < 0.001	
		24 (12M/12F)	296 ± 25	*p* < 0.001	
		18 (10M/8F)	409 ± 39	*p* = 0.005	
3B	Kruskal–Wallis + Dunn’s multiple comparisons	19 (10M/9F)	0.819 ± 0.015		H = 23,41
		19 (10M/9F)	0.709 ± 0.024	*p* = 0.005	
		24 (12M/12F)	0.838 ± 0.011	*p* < 0.001	
		18 (10M/8F)	0.844 ± 0.014	*p* < 0.001	
3C	One-way ANOVA + Dunnett’s multiple comparisons	19 (10M/9F)	80 ± 7		F (3, 77) = 17.81
		19 (10M/9F)	173 ± 11	*p* < 0.001	
		24 (12M/12F)	114 ± 9	*p* < 0.001	
		19 (11M/8F)	100 ± 9	*p* < 0.001	
4A	Welch ANOVA + Dunnett’s multiple comparisons	7 (4M/3F)	252 ± 43		10.98 (3.000, 15.40)
		7 (4M/3F)	480 ± 27	*p* = 0.003	
		10 (5M/5F)	352 ± 55	*p* = 0.152	
		10 (5M/5F)	285 ± 24	*p* < 0.001	
4B	One-way ANOVA + Dunnett’s multiple comparisons	7 (4M/3F)	0.820 ± 0.027		F (3, 30) = 8.189
		7(4M/3F)	0.602 ± 0.024	*p* < 0.001	
		10 (5M/5F)	0.741 ± 0.023	*p* = 0.006	
		10 (5M/5F)	0.737 ± 0.034	*p* = 0.008	
4C	Kruskal–Wallis + Dunn’s multiple comparisons	7 (4M/3F)	89 ± 11		H = 13,67
		7 (4M/3F)	151 ± 9	*p* = 0.028	
		10 (5M/5F)	96 ± 9	*p* = 0.020	
		10 (5M/5F)	82 ± 12	*p* = 0.001	
S1A	Two-way ANOVA + Dunnett’s multiple comparisons		Psilo 0–10 min	*p* < 0.001	
			Psilo 10–20 min	*p* < 0.001	
		6 (3M/3F)	Psilo 20–30 min	*p* = 0.001	
		6 (3M/3F)	DOI 0–10 min	*p* < 0.001	
		6 (3M/3F)	DOI 10–20 min	*p* < 0.001	
		6 (3M/3F)	DOI 20–30 min	*p* < 0.001	
			Lisu 0–10 min	*p* = 0.357	
			Lisu 10–20 min	*p* = 0.058	
			Lisu 20–30 min	*p* = 0.906	
S1B	Two-way ANOVA + Dunnett’s multiple comparisons		Psilo 0–10 min	*p* = 0.934	
		6 (3M/3F)	Psilo 10–20 min	*p* = 0.353	
		6 (3M/3F)	Psilo 20–30 min	*p* = 0.868	
		6 (3M/3F)	DOI 0–10 min	*p* = 0.607	
			DOI 10–20 min	*p* = 0.884	
			DOI 20–30 min	*p* > 0.999	
S1C	Two-way ANOVA + Dunnett’s multiple comparisons		Psilo 0–10 min	*p* = 0.092	
		6 (3M/3F)	Psilo 10–20 min	*p* = 0.205	
		6 (3M/3F)	Psilo 20–30 min	*p* = 0.321	
		6 (3M/3F)	DOI 0–10 min	*p* = 0.120	
			DOI 10–20 min	*p* = 0.234	
			DOI 20–30 min	*p* > 0.087	
S2A	One-way ANOVA + Dunnett’s multiple comparisons	9 (5M/4F)	321 ± 31		F (2, 26) = 6.428
		8 (4M/4F)	470 ± 46	*p* = 0.012	
		12 (7M/5F)	312 ± 26	*p* = 0.005	
S2B	One-way ANOVA + Dunnett’s multiple comparisons	9 (5M/4F)	0.788 ± 0.018		F (2, 26) = 9.691
		8 (4M/4F)	0.667 ± 0.032	*p* = 0.003	
		12 (7M/5F)	0.804 ± 0.019	*p* < 0.001	
S2C	Kruskal–Wallis + Dunn’s multiple comparisons	9 (5M/4F)	69 ± 11		H = 9906
		8 (4M/4F)	135 ± 18	*p* = 0.008	
		12 (7M/5F)	78 ± 7	*p* = 0.015	
		30 (16M/14F)	371 ± 21		
S2D	Kruskal–Wallis + Dunn’s multiple comparisons	28 (16M/12F)	560 ± 9	*p* < 0.001	H = 51,37
		24 (12M/12F)	296 ± 25	*p* < 0.001	
		12 (8M/4F)	352 ± 41	*p* < 0.001	
		12 (8M/4F)	386 ± 29	*p* < 0.001	
S2E	One-way ANOVA + Dunnett’s multiple comparisons	30 (16M/14F)	0.816 ± 0.013		F (4, 103) = 13.16
		30 (18M/12F)	0.691 ± 0.020	*p* < 0.001	
		24 (12M/12F)	0.838 ± 0.011	*p* < 0.001	
		12 (8M/4F)	0.795 ± 0.024	*p* = 0.002	
		12 (8M/4F)	0.788 ± 0.019	*p* = 0.003	
S2F	Kruskal–Wallis + Dunn’s multiple comparisons	30 (16M/14F)	84 ± 6		H = 46,38
		30 (18M/12F)	179 ± 9	*p* < 0.001	
		24 (12M/12F)	114 ± 9	*p* < 0.001	
		12 (8M/4F)	109 ± 9	*p* = 0.004	
		12 (8M/4F)	114 ± 11	*p* = 0.011	
S4A	One-way ANOVA + Dunnett’s multiple comparisons	20 (10M/10F)	373 ± 26		F (3, 45) = 2.273
		10 (5M/5F)	360 ± 41	*p* = 0.990	
		10 (5M/5F)	263 ± 25	*p* = 0.081	
		9 (5M/4F)	399 ± 59	*p* = 0.929	
S4B	Kruskal–Wallis + Dunn’s multiple comparisons	20 (10M/10F)	0.795 ± 0.016		H = 6386
		9 (5M/4F)	0.828 ± 0.019	*p* = 0.685	
		10 (5M/5F)	0.830 ± 0.015	*p* = 0.663	
		8 (5M/3F)	0.763 ± 0.018	*p* = 0.547	
S4C	Kruskal–Wallis + Dunn’s multiple comparisons	20 (10M/10F)	74 ± 9		H = 6177
		10 (5M/5F)	98 ± 15	*p* = 0.351	
		10 (5M/5F)	95 ± 12	*p* = 0.530	
		9 (5M/4F)	61 ± 9	*p* > 0.999	
S5A	Kruskal–Wallis + Dunn’s multiple comparisons	9 (5M/4F)	0.032 ± 0.004		H = 1090
		8 (4M/4F)	0.041 ± 0.007	*p* = 0.594	
		12 (7M/5F)	0.037 ± 0.005	*p* > 0.999	
S5B	Kruskal–Wallis + Dunn’s multiple comparisons	7 (4M/3F)	0.049 ± 0.009		H = 1309
		7 (4M/3F)	0.037 ± 0.007	*p* = 0.849	
		10 (5M/5F)	0.044 ± 0.007	*p* > 0.999	
		10 (5M/5F)	0.049 ± 0.009	*p* > 0.999	
S5C	Kruskal–Wallis + Dunn’s multiple comparisons	20 (10M/10F)	0.032 ± 0.005		H = 9378
		19 (10M/9F)	0.027 ± 0.005	*p* > 0.999	
		20 (11M/9F)	0.029 ± 0.003	*p* > 0.999	
		20 (10M/10F)	0.047 ± 0.006	*p* = 0.029	
		9 (5M/4F)	0.023 ± 0.004	*p* > 0.999	
S5D	One-way ANOVA + Dunnett’s multiple comparisons	19 (10M/9F)	0.049 ± 0.006		F (5, 98) = 2.274
		17 (8M/9F)	0.043 ± 0.005	*P* = 0.911	
		18 (10M/8F)	0.043 ± 0.006	*p* > 0.999	
		8 (4M/4F)	0.051 ± 0.008	*p* = 0.914	
		24 (12M/12F)	0.065 ± 0.006	*p* = 0.031	
		18 (10M/8F)	0.046 ± 0.006	*p* = 0.996	
S5E	Kruskal–Wallis + Dunn’s multiple comparisons	11 (6M/5F)	0.040 ± 0.008		H = 0,7129
		11 (8M/3F)	0.039 ± 0.006	*p* > 0.999	
		12 (8M/4F)	0.043 ± 0.003	*p* > 0.999	
		12 (8M/4F)	0.042 ± 0.005	*p* > 0.999	

### The antidepressant-like effects of DOI and lisuride are mediated by 5-HT_2A_R activation

**Fig. 2:**
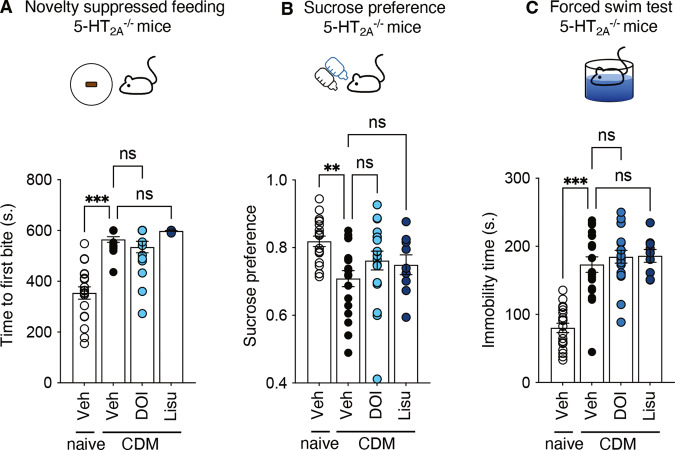
DOI and Lisuride do not produce antidepressant-like effects in 5-HT_2A_^−/−^ CDM mice. **A** The histogram represents the time to first bite (seconds) measured in the NSF paradigm for each condition. Naive mice + vehicle (*n* = 19), CDM mice + vehicle (*n* = 17), CDM mice + DOI (*n* = 18), CDM mice + lisuride (*n* = 8). ns, *p* > 0.05, ***p < 0.001 *vs*. CDM condition, Kruskal–Wallis test followed by Dunn’s test. **B** The histogram represents the sucrose preference index calculated for each condition. Naive mice + vehicle (*n* = 19), CDM mice + vehicle (*n* = 19), CDM mice + DOI (*n* = 19), CDM mice + lisuride (*n* = 9). ns, *p* > 0.05, **p < 0.01 *vs*. CDM condition, Kruskal–Wallis test followed by Dunn’s test. **C** The histogram represents the immobility time (seconds) measured in the FST for each condition. Naive mice + vehicle (*n* = 19), CDM mice + vehicle (*n* = 19), CDM mice + DOI (*n* = 19), CDM mice + lisuride (*n* = 9). ns, *p* > 0.05, ***p < 0.001 *vs*. CDM condition, one-way ANOVA test followed by Dunnett’s test. See Table 1 for mean ± SEM values.

To establish the role of 5-HT_2A_R activation in the antidepressant effect of the 5-HT_2A_R agonists tested, 5-HT_2A_^−/−^ mice were subjected to the chronic despair conditioning protocol (5-HT_2A_^−/−^ CDM mice) followed by the same behavioral tests. Similar to what was observed in wild-type CDM mice, the 5-HT_2A_^−/−^ CDM mice showed a depressive-like behavior compared to non-conditioned 5-HT_2A_^−/−^ mice (Fig. 2 and Table 1). Furthermore, the antidepressant-like effects induced by a single intraperitoneal injection of DOI (1 mg/kg) or lisuride (1 mg/kg) were abolished in 5-HT_2A_^−/−^ CDM mice (Fig. 2 and Table 1), indicating that they are mediated by 5-HT_2A_R activation.

### The antidepressant-like effects of psilocybin are independent of 5-HT_2A_, 5-HT_1A_, D1 and D2 receptor activation

**Fig. 3:**
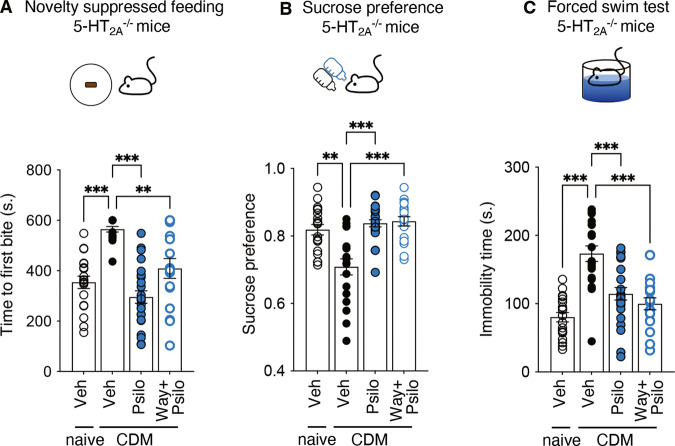
Psilocybin induces antidepressant-like effects independently of 5-HT_2A_R or 5-HT_1A_R stimulation. **A** The histogram represents the time to first bite (seconds) measured in the NSF paradigm for each condition in 5-HT_2A_^−/−^ mice. Naive mice + vehicle (*n* = 19), CDM mice + vehicle (*n* = 17), CDM mice + psilocybin (*n* = 24), CDM mice + WAY-100635 + psilocybin (*n* = 18). **p < 0.01, ***p < 0.001 *vs*. CDM condition, Kruskal–Wallis test followed by Dunn’s test. **B** The histogram represents the sucrose preference index calculated for each condition. Naive mice + vehicle (*n* = 19), CDM mice + vehicle (*n* = 19), CDM mice + psilocybin (*n* = 24), CDM mice + WAY-100635 + psilocybin (*n* = 18). **p < 0.01, ***p < 0.001 *vs*. CDM condition, Kruskal–Wallis test followed by Dunn’s test. **C** The histogram represents the immobility time (seconds) measured in the FST for each condition. Naive mice + vehicle (*n* = 19), CDM mice + vehicle (*n* = 19), CDM mice + psilocybin (*n* = 24), CDM mice + WAY100635+psilocybin (*n* = 19). ***p < 0.001 *vs*. CDM condition, one-way ANOVA test followed by Dunnett’s test. Note that naive and vehicle-injected CDM mice are the same as those used in experiments illustrated in Fig. 2A–C). See Table 1 for mean ± SEM values.

There are contradictory reports about the involvement of 5-HT_2A_Rs in the antidepressant properties of psilocybin [17, 18], the most widely studied psychedelic in clinical trials for its fast-acting antidepressant effects [5]. Reminiscent of its effects in wild type CDM mice, psilocybin administration (1 mg/kg) to 5-HT_2A_^−/−^ CDM mice significantly decreased both the latency to feed in the NSF test and the immobility time in the FST, compared to vehicle-injected mice (Fig. 3A, C and Table 1). Psilocybin also significantly increased sucrose preference in 5-HT_2A_^−/−^ CDM mice compared to vehicle-injected mice (Fig. 3B and Table 1). These results indicate that the antidepressant-like effects of psilocybin in CDM mice are independent of the 5-HT_2A_R.

Psilocin, the active metabolite of psilocybin, is a dual agonist of 5-HT_1A_ and 5-HT_2A_ receptors [31]. In order to determine the role of 5-HT_1A_R activation in the antidepressant-like effects of psilocybin, 5-HT_2A_^−/−^ CDM mice were injected with WAY-100635 (0.5 mg/kg, i.p.), a potent and selective 5-HT_1A_R antagonist, 30 min prior psilocybin administration. This treatment did not abolish the antidepressant-like effects of psilocybin in CDM mice (Fig. 3A–C and Table 1), suggesting that they are also independent of 5-HT_1A_R activation.

Previous studies have shown that a pretreatment with a D2 receptor antagonist reduces some of the positive symptoms induced by psilocybin intake in human [16, 32]. In order to explore the role of D1 and D2 dopamine receptor activation in the antidepressant-like effects of psilocybin, 5-HT_2A_^−/−^ CDM mice were injected either with SCH23390 (0.03 mg/kg, s.c.), a D1 receptor antagonist, or eticlopride, a selective D2 receptor antagonist (0.03 mg/kg, s.c.), 30 min prior to psilocybin administration. Neither SCH23390 nor eticlopride administration affected the antidepressant-like effects of psilocybin in CDM mice (Supplementary Fig. S2D–F and Table 1), suggesting that they are independent of D1 and D2 receptor activation.

### Sub-chronic microdosing administration of DOI or psilocybin induce antidepressant-like effects in CDM mice

**Fig. 4:**
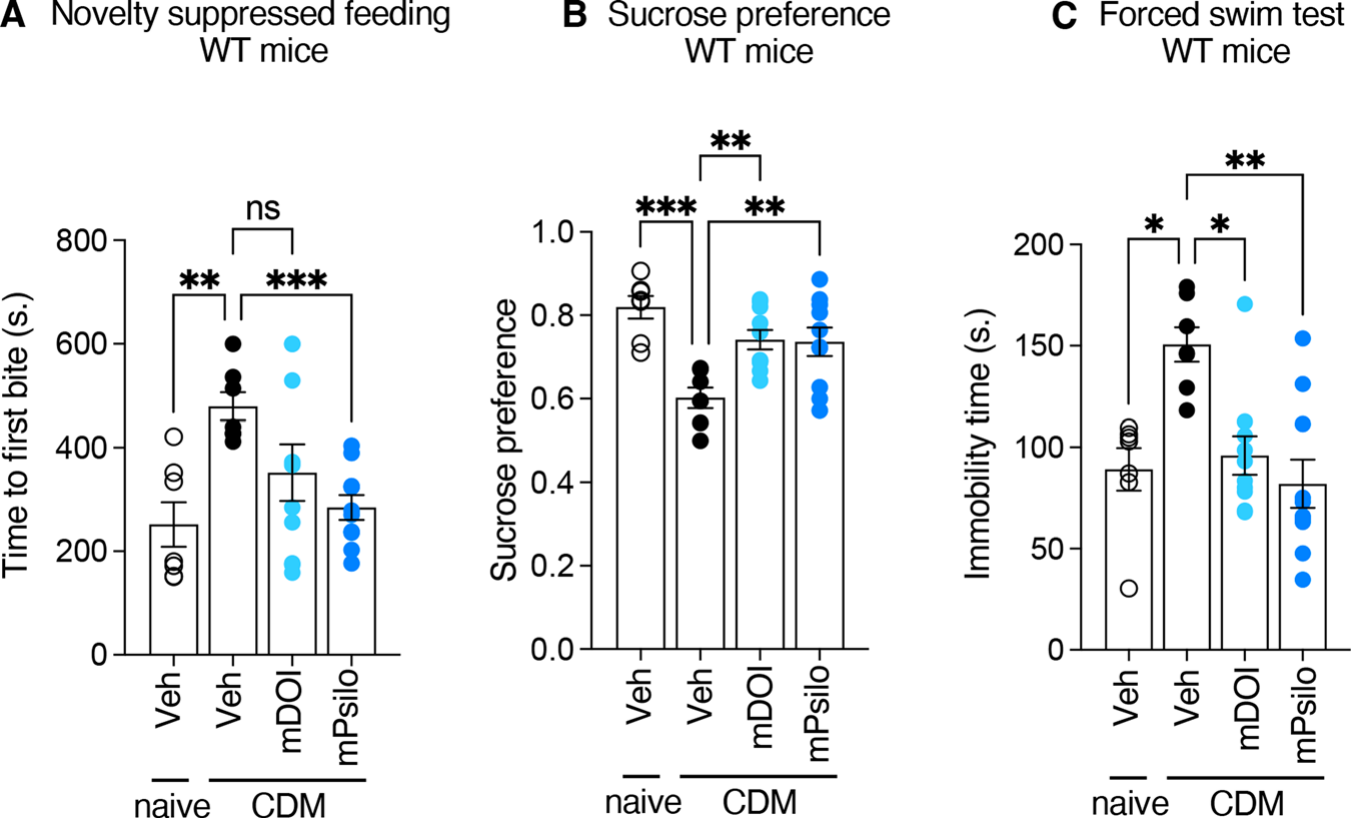
Sub-chronic microdosing administration of hallucinogenic 5-HT_2A_R agonists induces antidepressant-like effects in CDM mice. **A** The histogram represents the time to first bite (seconds) measured in the NSF paradigm for each condition. Naive mice + vehicle (*n* = 7), CDM mice + vehicle (*n* = 7), CDM mice + mDOI (*n* = 10), CDM mice + mPsilocybin (*n* = 10). ns, *p* > 0.05, **p < 0.01, ***p < 0.001  *vs*. CDM condition, Welch ANOVA test followed by Dunnett’s test. **B** The histogram represents the sucrose preference index calculated for each condition. Naive mice + vehicle (*n* = 7), CDM mice + vehicle (*n* = 7), CDM mice + mDOI (*n* = 10), CDM mice + mPsilocybin (*n* = 10). **p < 0.01, ***p < 0.001 *vs*. CDM condition, one-way ANOVA test followed by Dunnett’s test. **C** The histogram represents the immobility time (seconds) measured in the FST for each condition. Naive mice + vehicle (*n* = 7), CDM mice + vehicle (*n* = 7), CDM mice + mDOI (*n* = 10), CDM mice + mPsilocybin (*n* = 10). *p < 0.05, **p < 0.01 *vs*. CDM condition, Kruskal–Wallis test followed by Dunn’s test. See Table 1 for mean ± SEM values.

We next examined whether sub-chronic administration of sub-hallucinogenic doses of DOI or psilocybin (0.05 mg/kg each, i.p.) induce antidepressant-like effects in CDM mice. Drugs were injected daily for 6 days to mimic a “weekday” dosing. While these treatments did not induce a significant increase in HTRs, compared with vehicle-treated mice (Supplementary Fig. S1C and Table 1), they still produced antidepressant-like effects in the SPT and FST (Fig. 4B, C and Table 1). Likewise, psilocybin microdosing significantly decreased the latency to bite in the NSF test, but DOI microdosing only tended to decrease it (Fig. 4A and Table 1). Microdosing administration of the two drugs did not affect food consumption by CDM mice, as assessed immediately after the NSF test (Supplementary Fig. S5B and Table 1). Likewise, administration of a single dose of DOI or lisuride (1 mg/kg) did not alter food consumption of mice, whereas administration of 1 mg/kg psilocybin induced a significant increase in appetite of mice (Supplementary Fig. S5C and Table 1). In contrast to its antidepressant-like effects, the psilocybin-induced increase in food intake was prevented by the pretreatment of CDM mice with either WAY100635, or SCH23390 or eticlopride (Supplementary Fig. S5D, E and Table 1).

## Discussion

Although psychedelics activate many 5-HT receptors, including the 5-HT_1A_, 5-HT_2A_ and 5-HT_2C_ receptors [31], it is well accepted that the alterations of conscious states they induce in human are initiated by 5-HT_2A_R activation, as they are blocked by ketanserin, a potent 5-HT_2A_R antagonist [14, 16]. In addition, the psilocybin effects in human are correlated with the occupancy of 5-HT_2A_Rs in the cerebral cortex [33]. In contrast, the nature of the receptor implicated in the antidepressant effects of psychedelics remains controversial.

Here, we show that two psychedelics of different chemical families, DOI and psilocybin, exhibit similar efficiency to reverse depressive-like behaviors in CDM mice, as assessed in the NSF test, SPT and FST. The antidepressant-like effects of DOI were not observed in 5-HT_2A_^−/−^ CDM mice, indicating a prominent role of 5-HT_2A_Rs, as previously observed for its effects on extinction of fear memory [34], neuroplasticity (spine growth), expression of plasticity-related genes and synaptic plasticity [35–38]. In contrast, the antidepressant-like effects of psilocybin were still observed in CDM mice deficient in 5-HT_2A_R. This is consistent with the findings of Hesselgrave et al. [17] indicating that ketanserin does not inhibit psilocybin antidepressant-like effects in a model of chronic stress of similar etiology to the depression model used in the present study. The ability of ketanserin used at the same dose to inhibit the psilocybin-induced HTR in this study already suggested that the absence of effect of ketanserin on depressive symptoms was not related to the administration of an inadequate dose of antagonist. The lack of involvement of the 5-HT_2A_R in the antidepressant-like effects of psilocybin in chronic stress models of depression is also reminiscent of the antidepressant effects of ketamine that are independent of NMDA receptor inhibition [3]. Contrastingly, in a model of chronic corticosterone administration, Cameron et al. [18] reported that the antidepressant-like effects of psilocybin are abolished in 5-HT_2A_^−/−^ mice. These contradictory results may be due to the difference in the depression model used in both studies. The depressive-like states resulting from the chronic corticosterone treatment used by Cameron et al. and from the chronic stress used in our study might involve different neuronal networks and mechanisms. Accordingly, depression-like phenotype cannot be considered as a unique pathological state and it is likely that 5-HT_2A_Rs have a differential influence upon depressive symptoms depending on the etiology of the depressive-like state. The diversity of neuronal mechanisms underlying MDD of different etiologies might also explain why some patients do not respond to classical antidepressants or psilocybin treatment [10]. Another reason that might explain the discrepancies between our results and those of Cameron et al. [18] is the different mice strains used in both studies. Experiments described in the study of Hesselgrave et al. [17, 18] and the present report were performed on C57BL/6J mice, whereas Cameron et al. used the 129S6/SvEv mouse strain [18]. As stated by the authors, this strain does not respond to 5-MeO, a serotonergic psychedelic, or to ketamine in the FST, but does exhibit a depressive-like phenotype in the SPT test, which is a specificity of this model.

The identification of the receptor(s) and downstream mechanisms involved in the antidepressant-like effects of psilocybin in models of chronic stress certainly warrant further exploration. Given the agonist property of psilocin, the active metabolite of psilocybin, at 5-HT_1A_R and 5-HT_2A_R [31] and the antidepressant properties of 5-HT_1A_R agonists [39], we investigated the possibility that the antidepressant-like effects of psilocybin in 5-HT_2A_^−/−^ CDM mice involved the 5-HT_1A_R. However, WAY100635, a potent 5-HT_1A_R antagonist was totally ineffective to block psilocybin antidepressant-like effects, ruling out a role of 5-HT_1A_Rs in the effect of psilocybin in this model.

The use of psychedelics in psychiatric diseases is rapidly expanding in spite of some reservations about their clinical use at a large scale and the limitations of clinical studies assessing their efficiency. Double-blind clinical studies are almost impossible to carry on due to the obvious psychoactive effects of these drugs. In addition, psychedelic treatments are most often associated with a psychotherapy. It is thus difficult to define whether benefits must be attributed to psychedelics or the psychotherapy. Finally, the safety issues are difficult to refute in particular on the long-term and it is likely that illegal practice will take over. To circumvent these issues and in light of the increasing popularity of microdosing psychedelic intake due to the belief of its positive effects on mood state and some cognitive processes, such as attention [40], we investigated the effect of daily microdosing administration of psychedelics to CDM mice and showed that this protocol is as efficient as a single dose administration to induce antidepressant-like effects. Furthermore, this protocol did not result in an increased food consumption in psilocybin-treated mice, as observed after a single administration. This observation, together with the lack of HTR after sub-chronic microdosing psychedelic administration, supports its benefit over a single dose administration.

Recently, several groups of investigators have tested the idea that newly synthetized 5-HT_2A_R agonists devoid of psychedelic properties may still produce antidepressant effects [41–43], which would circumvent most of the aforementioned problems. Several newly synthesized compounds produced encouraging results in preclinical studies [42]. In this context, we examined whether lisuride, an ergot derivative behaving as a potent 5-HT_2A_R agonist and devoid of psychedelic action, induces antidepressant-like effects in CDM mice. Lisuride showed equivalent efficacy to DOI and psilocybin and its antidepressant effect was absent in 5-HT_2A_^−/−^ CDM mice. Interestingly, in another model of depression, lipopolysaccharide (LPS)-treated mice, lisuride administration also produced antidepressant-like effects [44], whereas DOI was ineffective [44]. Moreover, it was recently shown that Lisuride exerts antidepressant-like actions in VMAT2 (vesicular monoamine transporter 2) heterozygotes mice that exhibit anhedonia-like responses to sucrose solutions and enhanced immobility in tail suspension and forced swim tests [45]. Lisuride has been approved in Europe for more than 40 years for the treatment of parkinsonian symptoms and dyskinesias [27] or to lower prolactin level [28]. Even if it is currently less prescribed due to its lower effectiveness on Parkinson symptoms compared to other D2 receptor agonists, it has a wide margin of safety. Our results showing a robust antidepressant-like effect of lisuride without inducing HTR in CDM mice also support the notion that a psychedelic experience might not be required for the therapeutic response to 5-HT_2A_R agonists [46]. Consistent with this hypothesis, a recent case report showed that the antidepressant effect of psilocybin is independent of 5-HT_2A_R activation and, accordingly, of its psychedelic activity [47]. However, another report from patients/volunteers showing that the altered state of consciousness produced by psychedelics is critical for their clinical efficacy [48] indicates that the role of psychedelic properties of 5-HT_2A_R agonists in their antidepressant effects remains an open question. Even though it is difficult to predict therapeutic efficacy of psychedelics in patients from their pharmacological effects in preclinical models, the robust antidepressant-like effects of lisuride in CDM mice also indicate that clinical trials investigating its antidepressant action in patients with MDD and resistant to conventional treatments would certainly be of great interest.

### Supplementary information

Supplementary figure

Supplementary table
