# Supplementary material for: Antidepressant-like effects of psychedelics in a chronic despair mouse model: is the 5-HT2A receptor the unique player?
Source: Neuropsychopharmacology. 2024 Jan 11;49(4):747–56. doi: 10.1038/s41386-024-01794-6 (PMC10876623; doi:10.1038/s41386-024-01794-6)
Supplement: Supplementary file 1 — Supplementary figure [file 41386_2024_1794_MOESM1_ESM.pdf]

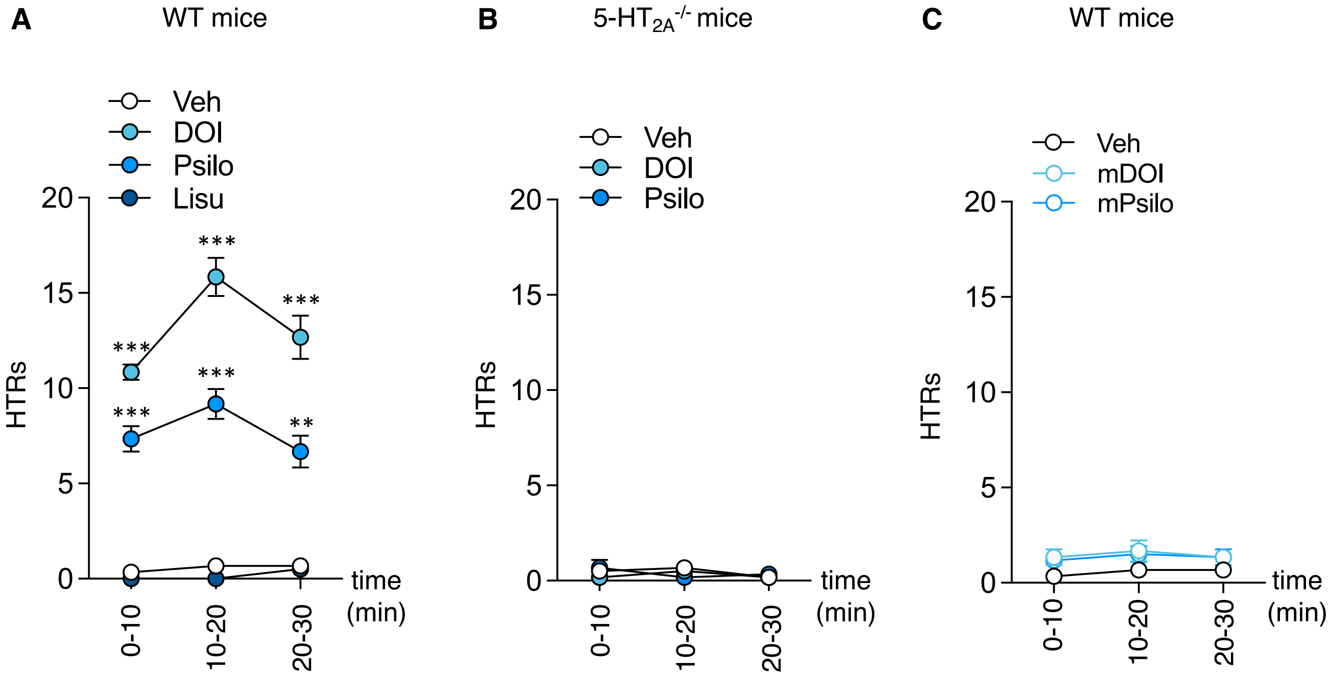

A
WT mice
Veh
DOI
Psilo
Lisu
HTRs
20
15
10
5
0
***
***
***
***
***
**
time
(min)
0-10
10-20
20-30
B
5-HT$_{2A}^{-/-}$ mice
Veh
DOI
Psilo
HTRs
20
15
10
5
0
time
(min)
0-10
10-20
20-30
C
WT mice
Veh
mDOI
mPsilo
HTRs
20
15
10
5
0
time
(min)
0-10
10-20
20-30

Figure S1

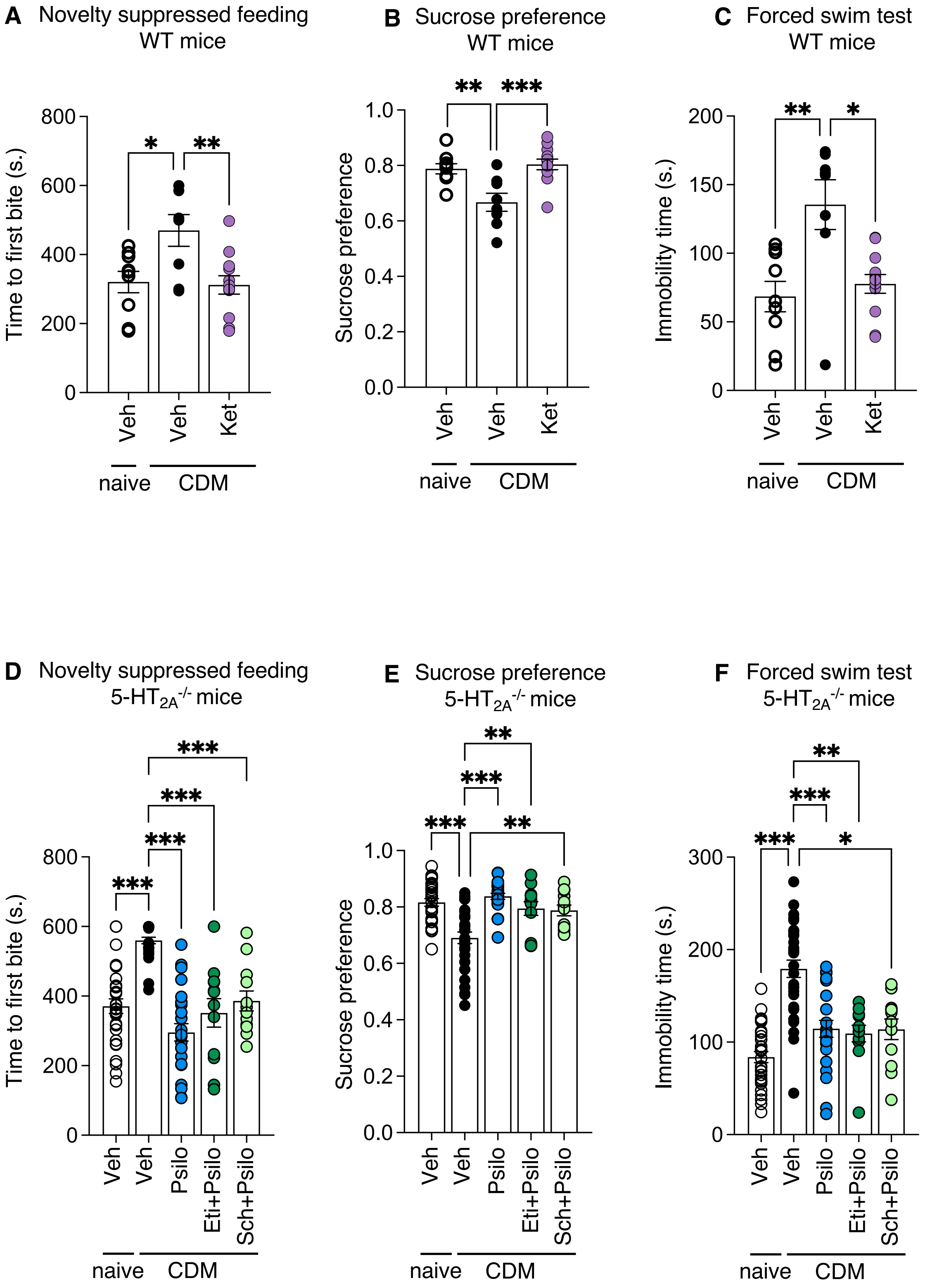

A Novelty suppressed feeding WT mice
Time to first bite (s.)
Veh
Veh
Ket
naive
CDM
B Sucrose preference WT mice
Sucrose preference
Veh
Veh
Ket
naive
CDM
C Forced swim test WT mice
Immobility time (s.)
Veh
Veh
Ket
naive
CDM
D Novelty suppressed feeding 5-HT2A-/- mice
Time to first bite (s.)
Veh
Veh
Psilo
Eti+Psilo
Sch+Psilo
naive
CDM
E Sucrose preference 5-HT2A-/- mice
Sucrose preference
Veh
Veh
Psilo
Eti+Psilo
Sch+Psilo
naive
CDM
F Forced swim test 5-HT2A-/- mice
Immobility time (s.)
Veh
Veh
Psilo
Eti+Psilo
Sch+Psilo
naive
CDM

Figure S2

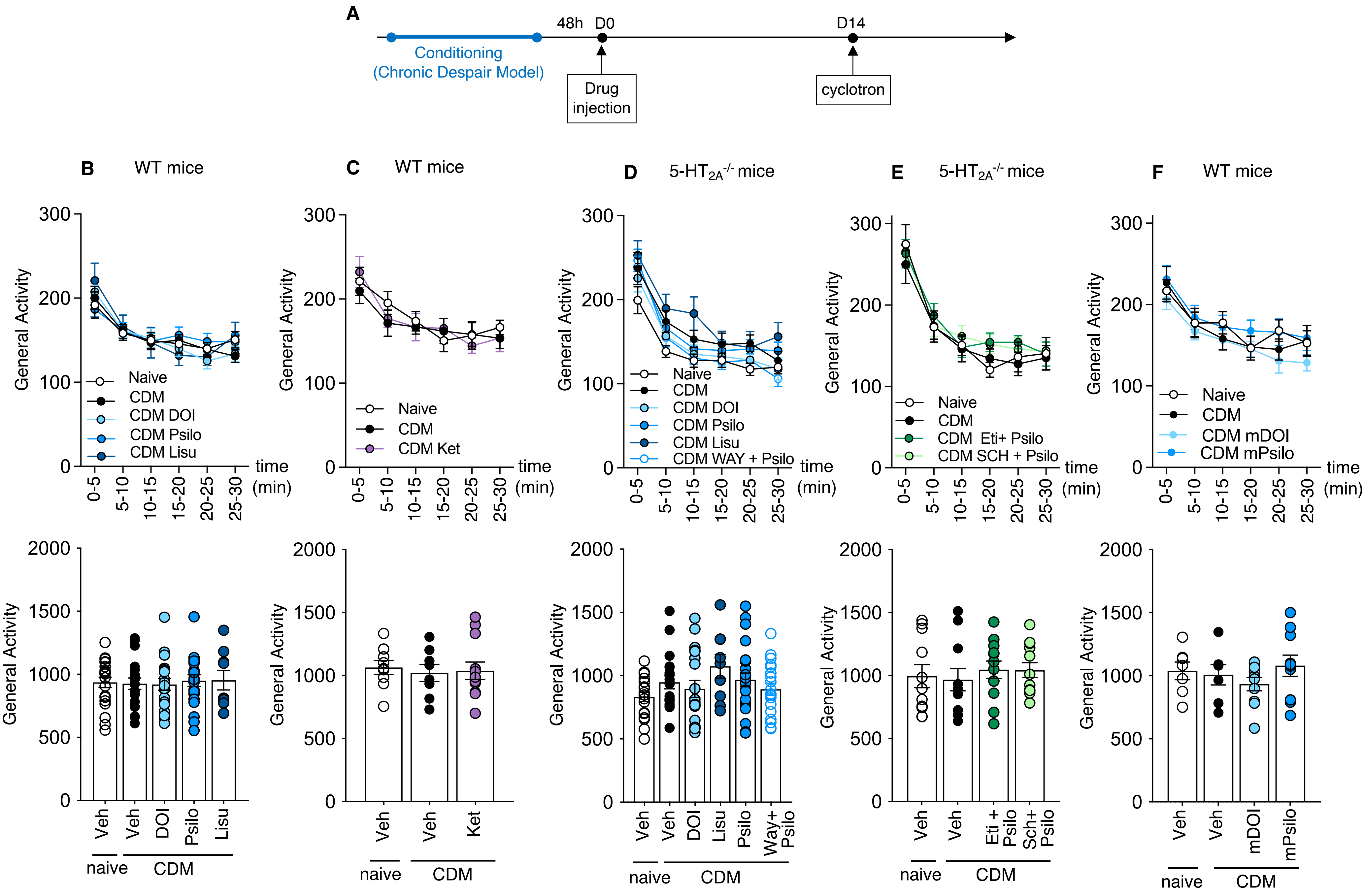
A
48h
D0
D14
Conditioning
(Chronic Despair Model)
Drug injection
cyclotron
B
WT mice
C
WT mice
D
5-HT2A-/- mice
E
5-HT2A-/- mice
F
WT mice
General Activity
time (min)
0-5
5-10
10-15
15-20
20-25
25-30
Naive
CDM
CDM DOI
CDM Psilo
CDM Lisu
CDM Ket
CDM WAY + Psilo
CDM Eti+ Psilo
CDM SCH + Psilo
CDM mDOI
CDM mPsilo
Veh
DOI
Psilo
Lisu
Ket
Way+ Psilo
Eti + Psilo
Sch+ Psilo
mDOI
mPsilo
naive
CDM

Figure S3

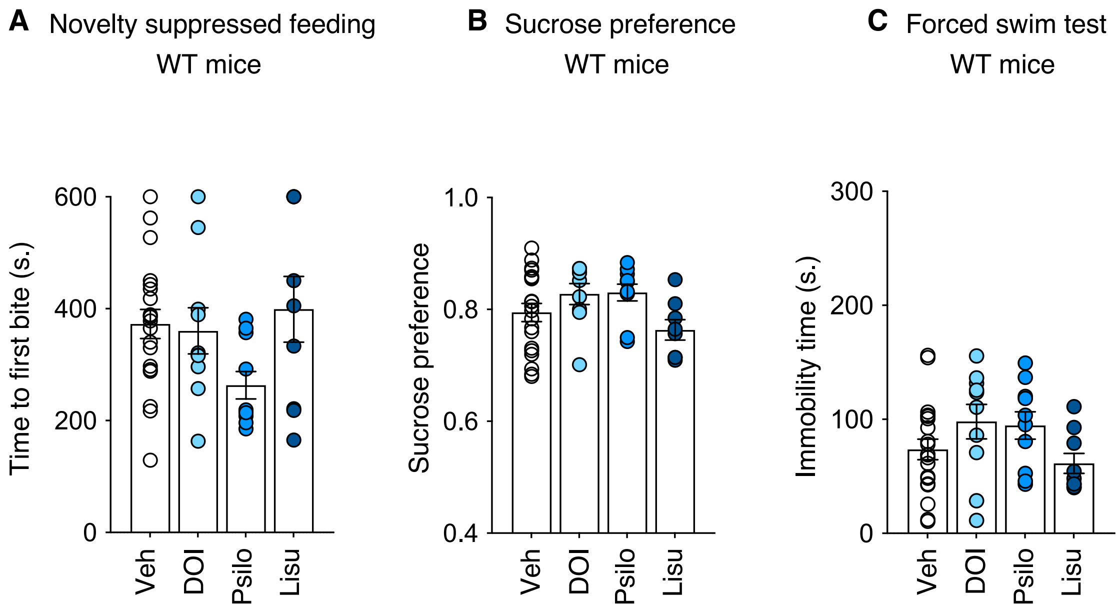
A Novelty suppressed feeding
WT mice
Time to first bite (s.)
600
400
200
0
Veh
DOI
Psilo
Lisu
B Sucrose preference
WT mice
Sucrose preference
1.0
0.8
0.6
0.4
Veh
DOI
Psilo
Lisu
C Forced swim test
WT mice
Immobility time (s.)
300
200
100
0
Veh
DOI
Psilo
Lisu

Figure S4

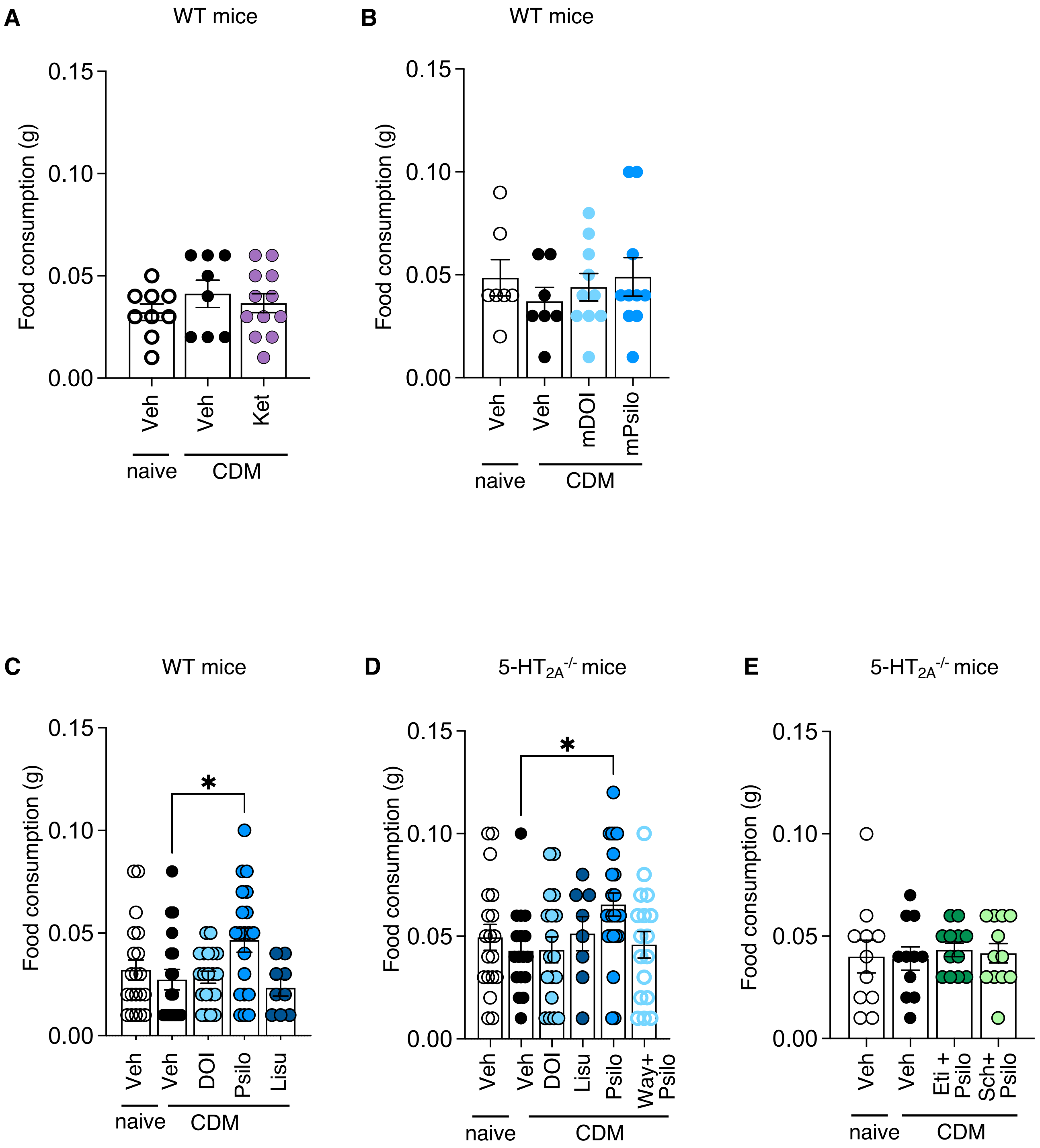

A
WT mice
B
WT mice
C
WT mice
D
5-HT2A-/- mice
E
5-HT2A-/- mice
Food consumption (g)
0.15
0.10
0.05
0.00
Veh
Ket
mDOI
mPsilo
DOI
Psilo
Lisu
Way+ Psilo
Eti + Psilo
Sch+ Psilo
naive
CDM
*

Figure S5

Fig S1: Hallucinogenic 5-HT$_{2A}$R agonists produce head twitch responses in wild type but not in 5-HT$_{2A}$$^{-/-}$ mice or after micro-dosing administration of psychedelics to wild type mice.

A: The graphic represents the number of head twitches measured for 30 min during 10-min time frames in wild type mice. Naive mice + vehicle (n=6), naive mice + DOI (n=6), naive mice + Psilocybin (n=6), naive mice + Lisuride (n=6). ns, $p > 0.05$, ** $p < 0.01$, *** $p < 0.001$ *vs.* naive mice + vehicle condition, two-way ANOVA test followed by Dunnett's test. **B:** The data represent the number of head twitches measured for 30 min during 10-min time frames in 5-HT$_{2A}$$^{-/-}$ mice. Naive mice + vehicle (n=6), naive mice + DOI (n=6), naive mice + psilocybin (n=6). ns, $p > 0.05$, *vs.* naive mice + vehicle condition, two-way ANOVA test followed by Dunnett's test. **C:** The graphic represents the number of head twitches measured for 30 min during 10-min time frames in wild type mice. Naive mice + vehicle (n=6), naive mice + micro-dosing DOI (mDOI, n=6), naive mice + micro-dosing psilocybin (mPsilo, n=6). ns, $p > 0.05$ *vs.* naive mice + vehicle condition, two-way ANOVA test followed by Dunnett's test. Note that vehicle-injected mice are the same as those used in experiments illustrated on Figure S1A. See Table 1 for mean ± SEM values.

Fig S2: Ketamine produces antidepressant-like effects in CDM mice and psilocybin acts independently of either D$_1$ or D$_2$ receptors.

A: The histogram represents the time to first bite (seconds) measured in the NSF paradigm for each condition. Naive mice + vehicle (n=9), CDM mice + vehicle (n=8), CDM mice + ketamine (n=12). * $p < 0.05$, ** $p < 0.01$ *vs.* CDM condition, one-way ANOVA test followed by Dunnett's test. **B**: The histogram represents the sucrose preference index calculated for each condition. Naive mice + vehicle (n=9), CDM mice + vehicle (n=8), CDM mice + ketamine (n=12), ** $p < 0.01$, *** $p < 0.001$ *vs.* CDM condition, one-way ANOVA test followed by

Dunnett's test. **C**: The histogram represents the immobility time (seconds) measured in the FST for each condition. Naive mice + vehicle (n=9), CDM mice + vehicle (n=8), CDM mice + ketamine (n=12). * $p < 0.05$, ** $p < 0.01$ *vs.* CDM condition, Kruskal-Wallis test followed by Dunn's test. **D**: The histogram represents the time to first bite (seconds) measured in the NSF paradigm for each condition. Naive mice + vehicle (n=30), CDM mice + vehicle (n=28), CDM mice + psilocybin (n=24), CDM mice + eticlopride + psilocybin (n=12), CDM mice + SCH23390 + Psilocybin (n=12). *** $p < 0.001$ *vs.* CDM condition, Kruskal-Wallis test followed by Dunn's test. **E**: The histogram represents the sucrose preference index calculated for each condition. Naive mice + vehicle (n=30), CDM mice + vehicle (n=30), CDM mice + psilocybin (n=24), CDM mice + eticlopride + psilocybin (n=12), CDM mice + SCH23390 + Psilocybin (n=12). ** $p < 0.01$, *** $p < 0.001$ *vs.* CDM condition, one-way ANOVA test followed by Dunnett's test. **F**: The histogram represents the immobility time (seconds) measured in the FST for each condition. Naive mice + vehicle (n=30), CDM mice + vehicle (n=30), CDM mice + psilocybin (n=24), CDM mice + eticlopride + psilocybin (n=12), CDM mice + SCH23390 + Psilocybin (n=12). * $p < 0.05$, ** $p < 0.01$, *** $p < 0.001$ *vs.* CDM condition, Kruskal-Wallis test followed by Dunn's test. Note that naïve, vehicle-injected CDM mice and psilocybin-injected CDM mice from Figures 3A-C were pooled in this graph. See Table 1 for mean ± SEM values.

Fig S3: Hallucinogenic and non-hallucinogenic 5-HT$_{2A}$R agonists do not modify locomotor activity in naive and CDM wild type and 5-HT$_{2A}^{-/-}$ mice.

A: Timeline of treatment and behavioral experiments. Mice received a single injection of the tested compounds 48 h after the end of the chronic despair protocol. The locomotor activity of mice was measured in the cyclotron 14 days after the injection of the drug (Day 0). **B:** The graphic represents the locomotor activity of wild type mice measured for 30 min in each

condition. Data are categorized in 5-min time frames (upper panel). Naive mice + vehicle (n=20), CDM mice + vehicle (n=19), CDM mice + DOI (n=20), CDM mice + psilocybin (n=20), CDM mice + lisuride (n=9). $p > 0.05$ *vs.* CDM condition, two-way ANOVA test followed by Dunnett's test (upper panel) or one-way ANOVA test followed by Dunnett's test (bottom panel). **C:** The graphic represents the locomotor activity of wild type mice measured for 30 min in each condition. Data are categorized in 5 min frames (upper panel). Naive mice + vehicle (n=9), CDM mice + vehicle (n=8), CDM mice + Ketamine (n=12). $p > 0.05$ *vs.* CDM condition, two-way ANOVA test followed by Dunnett's test (upper panel) or one-way ANOVA test followed by Dunnett's test (bottom panel). **D:** The graphic represents the locomotor activity of wild type mice measured for 30 min in each condition. Data are categorized in 5 min frames (upper panel). Naive mice + vehicle (n=19), CDM mice + vehicle (n=19), CDM mice + DOI (n=19), CDM mice + psilocybin (n=24), CDM mice + lisuride (n=9), CDM mice + WAY-100635 + Psilocybin (n=19). $p > 0.05$ *vs.* CDM condition, two-way ANOVA test followed by Dunnett's test (upper panel) or Kruskal-Wallis test followed by Dunn's test (bottom panel). **E:** The graphic represents the locomotor activity of wild type mice measured for 30 min in each condition. Data are categorized in 5 min frames (upper panel). Naive mice + vehicle (n=11), CDM mice + vehicle (n=11), CDM mice + eticlopride + Psilocybin (n=12), CDM mice + SCH-23390 + Psilocybin (n=12). $p > 0.05$ *vs.* CDM condition, two-way ANOVA test followed by Dunnett's test (upper panel) or one-way ANOVA test followed by Dunnett's test (bottom panel). **F:** The graphic represents the locomotor activity of wild type mice measured for 30 min in each condition. Data are categorized in 5 min frames (upper panel). Naive mice + vehicle (n=7), CDM mice + vehicle (n=7), CDM mice + mDOI (n=10), CDM mice + mPsilocybin (n=10). $p > 0.05$ *vs.* CDM condition, two-way ANOVA test followed by Dunnett's test (upper panel) or one-way ANOVA test followed by Dunnett's test (bottom panel). See Table S1 for p values.

Fig S4: Hallucinogenic or non-hallucinogenic 5-HT$_{2A}$R agonists do not affect depressive-like behaviors in naive mice

A: The histogram represents the time to first bite (seconds) measured in the NSF paradigm for each condition. Naive mice + vehicle (n=20), + DOI (n=10), + psilocybin (n=10), + lisuride (n=9). $p > 0.05$ *vs.* Veh condition, one-way ANOVA test followed by Dunnett's test. **B:** The histogram represents the sucrose preference index calculated for each condition. Naive mice + vehicle (n=20), + DOI (n=9), + psilocybin (n=10) and + lisuride (n=8). $p > 0.05$ *vs.* Veh condition, Kruskal-Wallis test followed by Dunn's test. **C:** The histogram represents the immobility time (s) measured in the FST for each condition. Naive mice + vehicle (n=20), + DOI (n=10), + psilocybin (n=10), and + lisuride (n=9). $p > 0.05$ *vs.* Veh condition, Kruskal-Wallis test followed by Dunn's test. Note that vehicle-injected mice are the same as those used in experiments illustrated in Figures 1B–D). See Table 1 for mean ± SEM values.

Fig S5: Food consumption assessment following the NSF test.

A: The histogram represents the food consumption (g) measured in the NSF paradigm during 5 min for each condition. Naive mice + vehicle (n=9), CDM mice + vehicle (n=8), CDM mice + ketamine (n=12). ns, $p > 0.05$ *vs.* CDM condition, Kruskal-Wallis test followed by Dunn's test. **B:** The histogram represents the food consumption (g) measured in the NSF paradigm during 5 min for each condition. Naive mice + vehicle (n=7), CDM mice + vehicle (n=7), CDM mice + mDOI (n=10), CDM mice + mpsilocybin (n=10). $p > 0.05$ *vs.* CDM condition, Kruskal-Wallis test followed by Dunn's test. **C:** The histogram represents the food consumption (g) measured in the NSF paradigm during 5 min for each condition. Naive mice + vehicle (n=20), CDM mice + vehicle (n=19), CDM mice + DOI (n=20), CDM mice + psilocybin (n=20), CDM mice + lisuride (n=9). ns, $p > 0.05$, * $p < 0.05$ *vs.* CDM condition, Kruskal-Wallis test followed

by Dunn’s test. **D:** The histogram represents the food consumption (g) measured in the NSF paradigm during 5 min for each condition in 5-$HT_{2A}^{-/-}$ mice. Naive mice + vehicle (n=19), CDM mice + vehicle (n=17), CDM mice + DOI (n=18), CDM mice + lisuride (n=8), CDM mice + psilocybin (n=24), CDM mice + WAY-100635 + psilocybin (n=18). ns, $p > 0.05$, * $p < 0.05$ *vs.* CDM condition, one-way ANOVA test followed by Dunnett’s test. **E:** The histogram represents the food consumption (g) measured in the NSF paradigm during 5 min for each condition in 5-$HT_{2A}^{-/-}$ mice. Naive mice + vehicle (n=11), CDM mice + vehicle (n=11), CDM mice + eticlopride + psilocybin (n=12), CDM mice + SCH23390 + psilocybin (n=12). $p > 0.05$ *vs.* CDM condition, Kruskal-Wallis test followed by Dunn’s test. See Table 1 for mean ± SEM values.

Table S1: Mean ± SEM, n and p values related to Figure S3.
